# Toxic Mechanism of Norfloxacin on *Chlamydomonas reinhardtii* by Triggering Programmed Cell Death

**DOI:** 10.3390/plants15071015

**Published:** 2026-03-26

**Authors:** Xianmin Du, Lexin Huang, Meng Lai, Haozhe Xu, Tianyu Huang, Rong Hu, Junjie Ma, Yinggang Wei, Zhaojiang Zuo

**Affiliations:** 1 College of Forestry and Biotechnology, Zhejiang A&F University, Hangzhou 311300, China; 2 Zhejiang Provincial Key Laboratory of Forest Aromatic Plants-based Healthcare Functions, Zhejiang A&F University, Hangzhou 311300, China

**Keywords:** cell morphology, *Chlamydomonas reinhardtii*, mitochondrial membrane potential, norfloxacin, programmed cell death

## Abstract

Norfloxacin has been widely found in water bodies and exhibits a strong toxic effect on aquatic organisms. To uncover its toxic mechanism on algae, the cell growth, reactive oxygen species (ROS) levels, physiological activities, mitochondrial membrane potential (MMP), caspase-3-like activity, cell morphology, TUNEL-positive nuclei and DNA ladders were determined in *Chlamydomonas reinhardtii* in exposure to norfloxacin. With raising norfloxacin concentration, the inhibitory and lethal effects on *C. reinhardtii* cells gradually enhanced, and the whole of the cells were dead under 50 μM for 24 h. During the cell death, respiratory and photosynthetic rate gradually reduced and disappeared after 24 h, while ROS quickly burst and maintained high levels during the 24 h. The MMP was markedly broken after 0.5 h, while caspase-3-like was activated, with the highest activity at the 2nd h. With prolonging the treatment time, the algal cells showed a gradual shrinking and wrinkling trend, while the numbers and fluorescence intensity of TUNEL-positive nuclei gradually increased. Meanwhile, the DNA was degraded by Ca^2+^-dependent endonucleases to show ladders after 6 h, and the degradation gradually enhanced during the death process. These characteristics demonstrate that norfloxacin can poison algae by triggering programmed cell death induced by the elevated ROS.

## 1. Introduction

Antibiotics are widely used in medicine, agricultural production, animal husbandry, aquaculture and other fields to inhibit the growth and reproduction of microorganisms, with the consumption of 100,000 to 200,000 tons annually worldwide [1]. However, the widespread usage of antibiotics has led to serious environmental pollution [2]. This pollution is primarily caused by the discharge of medical wastewater, misuse of antibiotics in farming practices, improper antibiotic production and waste disposal [3]. In water bodies, the antibiotic concentration can range from ng·L^−1^ to mg·L^−1^ [4]. For instance, the total concentration of antibiotics in coastal waters of China ranges from 389 to 3302.3 ng·L^−1^ [5].

Norfloxacin is a broad-spectrum quinolone antibiotic, which is known for the significant antibacterial activity by inhibiting DNA replication and DNase activity [6,7]. In waters, norfloxacin is mainly derived from the discharge of domestic, medical, livestock and aquaculture wastewater, and is either free form or binds with suspended particles [8]. In aquaculture waters and waste waters, the high concentration of norfloxacin can reach 982 (3.1 μM) and 420 (1.3 μM) μg·L^−1^, respectively [4,9]. For another quinolone antibiotic ciprofloxacin, its concentration in waste waters even arrived at 31,000 μg·L^−1^ (93.6 μM) [9].

In water bodies, the presence of norfloxacin poses a serious threat to aquatic organisms [10]. For fishes, norfloxacin not only exhibits genotoxicity, neurotoxicity and immunotoxicity, but also affects the embryonic development [11]. In exposure to the antibiotic, several important pathways such as energy metabolism, amino acid metabolism, neuro-regulation and osmotic pressure regulation in *Mytilus* sp. were disturbed [12]. For *Daphnia magna*, norfloxacin can influence its adaptability to environmental variations by inhibiting the heartbeat and locomotion ability, feeding capacity, as well as population dynamics [13]. Except for aquatic animals, aquatic plants are also impacted by the antibiotic, e.g., norfloxacin caused toxic effects on *Lemna minor*, *Spirodela polyrrhiza* and *Vallisneria natans* by inhibiting the growth, increasing reactive oxygen species (ROS) accumulation, intensifying membrane lipid peroxidation, and destroying photosynthetic systems [10,14,15].

Algae and cyanobacteria are essential primary producers in aquatic ecosystems and play fundamental roles in maintaining the ecosystem structure and function [16]. As photosynthetic prokaryotes, cyanobacteria are sensitive to norfloxacin, due to their similarity with bacteria. When *Microcystis aeruginosa* cells were exposed to norfloxacin, their growth, chlorophyll synthesis, photosynthesis and primary productivity were inhibited [17], ROS were accumulated to high levels with causing strong oxidative stress [10], and DNA replication and transcription as well as mRNA translation were blocked [18]. Similarly, norfloxacin also inhibited the cell growth and photosynthetic abilities in other cyanobacteria, such as *Chrysosporum ovalisporum* [19] and *Arthrospira platensis* [20].

Although eukaryotic algae have a different cellular structure with cyanobacteria, norfloxacin also exhibit toxic effects on them, e.g., the antibiotic remarkedly inhibited the growth and reproduction of *S. obliquus*, *Dunaliella salina*, *Selenastrum capricornutum* and *Chlorella vulgaris* [13,21,22,23]. In exposure to norfloxacin, ROS were excessively accumulated in *S. obliquus* and *Chlorella* sp., resulting in damages of membrane systems and reduction of metabolic processes [24,25]. For *Scenedesmus quadricauda* and *S. obliquus*, norfloxacin can impact their colony formation [13,26], which may influence phytoplankton interactions and plankton ecosystem stability [27,28,29]. Moreover, the lethal effect of norfloxacin was also detected in some algae, such as *Pseudokirchneriella subcapitata* [30], *C. vulgaris* [31] and *S. obliquus* [25]. However, the lethal mechanism is still unknown.

*Chlamydomonas reinhardtii* is a primary green alga in worldwide freshwaters and is widely used in toxicological and biological studies as a model organism [32,33,34,35]. In this study, the toxic effect of norfloxacin on the alga has been investigated, and the toxic mechanism has been uncovered by detecting ROS levels, photosynthetic and respiratory rate, mitochondrial membrane potential (MMP), caspase-3-like activity, cell morphology, TUNEL-positive nuclei and DNA ladders. As far as we know, this is the first report about norfloxacin lethal mechanism on algae by causing programmed cell death (PCD), which is beneficial to uncovering norfloxacin toxic mechanism on aquatic organisms and providing help for environmental protection and antibiotic governance.

## 2. Results

### 2.1. Suppression of Cell Growth

**Figure 1:**
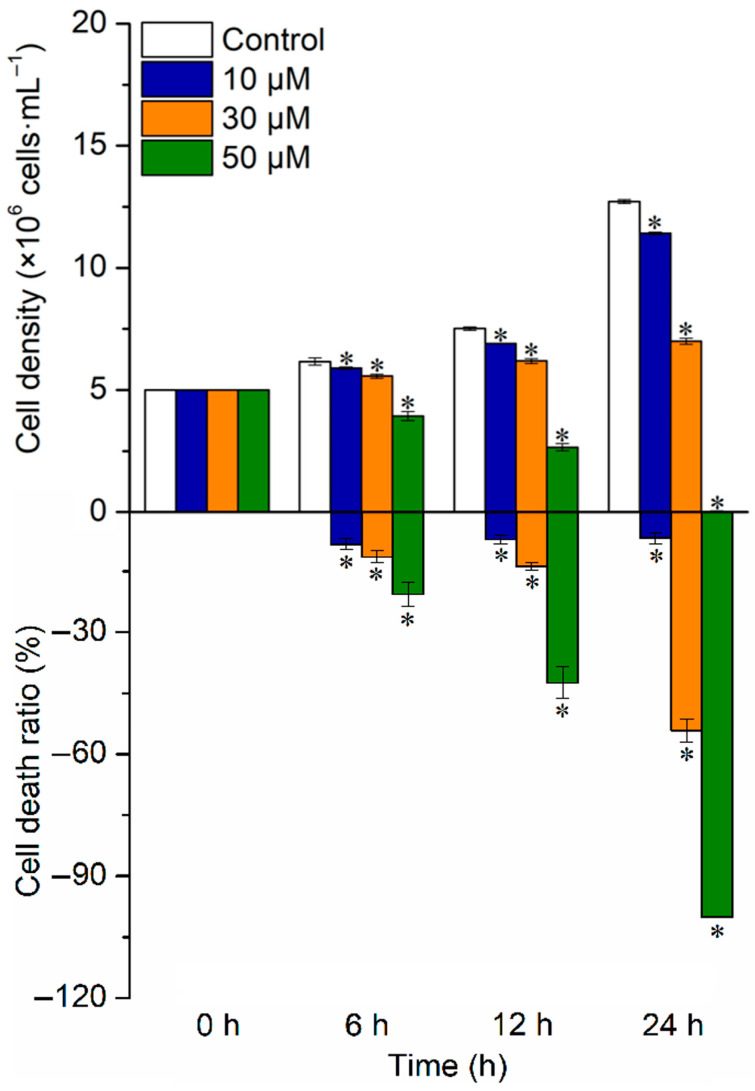
Effects of norfloxacin on *C. reinhardtii* cell growth. *: Significant difference at *p* < 0.05 compared with the control (without norfloxacin treatment). Negative value means the dead cells. Means ± SE (*n* = 4).

When *C. reinhardtii* cells were exposed to norfloxacin, the inhibition of cell growth gradually intensified with raising the antibiotic concentration, and the dead ratio also gradually increased. Meanwhile, the inhibition and dead ratio in 30 and 50 μM norfloxacin treatments gradually intensified with prolonging treatment time. After 24 h, the cell density decreased by 10.2% (*p* < 0.05), 49.9% (*p* < 0.05) and 100% (*p* < 0.05) under 10, 30 and 50 μM norfloxacin, respectively, while the dead ratio reached 6.8% (*p* < 0.05), 54.1% (*p* < 0.05) and 100% (*p* < 0.05), respectively (Figure 1).

### 2.2. Increase in ROS Levels

**Figure 2:**
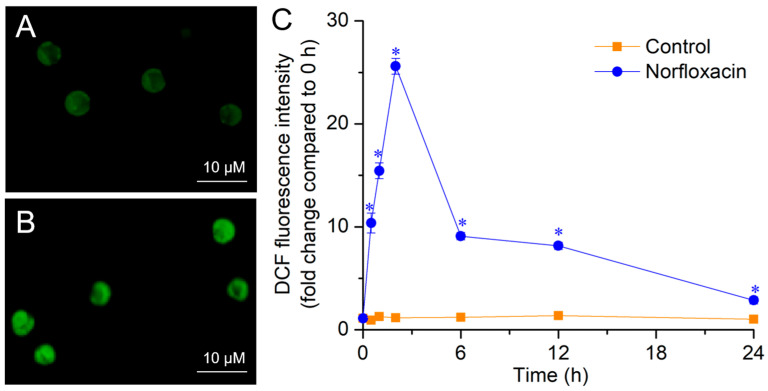
Effects of 50 μM norfloxacin on ROS levels in *C. reinhardtii*. (**A**,**B**) Fluorescence image in the control (without norfloxacin treatment) and 50 μM norfloxacin treatment for 2 h, respectively; (**C**) Fluorescence intensity of 2′,7′-dichlorofluorescein (DCF). *: Significant difference at *p* < 0.05 compared with the control. Means ± SE (*n* = 4).

To uncover the lethal mechanism of norfloxacin on *C. reinhardtii* cells, some related indexes were measured in the treatment with the concentration of 50 μM due to the 100% lethal ratio. In exposure to 50 μM norfloxacin, the ROS content in *C. reinhardtii* cells was significantly (*p* < 0.05) higher than the control. After 2 h, the algal cells accumulated ROS to the maximum level, showing bright green fluorescence, and the ROS levels were 21.9-fold higher than the control (Figure 2).

### 2.3. Decrease in Photosynthetic and Respiratory Rate

**Figure 3:**
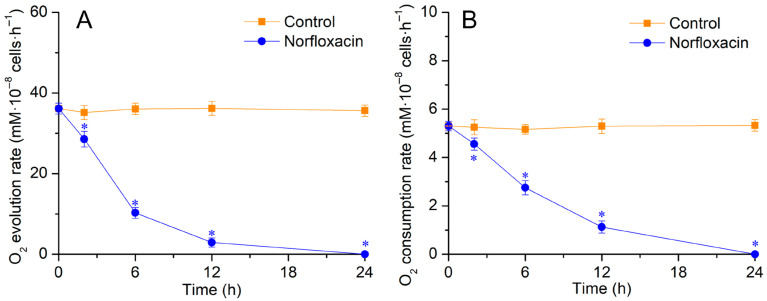
Effects of 50 μM norfloxacin on O_2_ evolution (**A**) and consumption (**B**) rate in *C. reinhardtii*. *: Significant difference at *p* < 0.05 compared with the control (without norfloxacin treatment). Means ± SE (*n* = 4).

In the treatment with 50 μM norfloxacin for 2 h, the O_2_ evolution and consumption rate in *C. reinhardtii* cells declined by 18.8% (*p* < 0.05) and 13.1% (*p* < 0.05), respectively. Then, they gradually decreased with prolonging treatment time, and finally disappeared after 24 h, indicating that the physiological activities gradually disappeared during the cell death (Figure 3).

### 2.4. MMP Decrease

**Figure 4:**
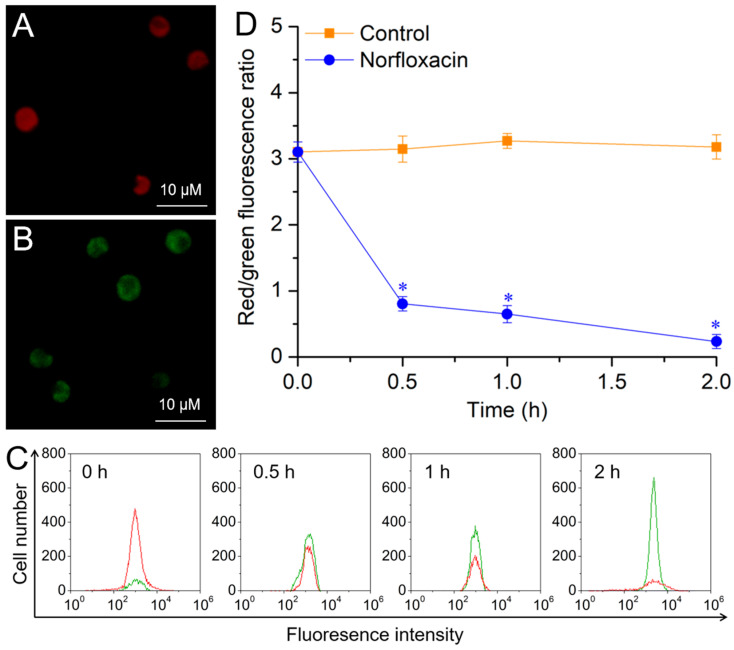
Effects of 50 μM norfloxacin on mitochondrial membrane potential in *C. reinhardtii*. (**A**,**B**) Fluorescence image in the control (without norfloxacin treatment) and 50 μM norfloxacin treatment for 2 h, respectively. (**C**) Red and green fluorescence peaks. (**D**) Red/green fluorescence ratio. *: Significant difference at *p* < 0.05 compared with the control. Means ± SE (*n* = 4).

For the control, *C. reinhardtii* cells contained healthy mitochondria and mainly showed red fluorescence observed with a fluorescence microscope (Figure 4A). During the 2 h treatment with 50 μM norfloxacin, the red fluorescence intensity (red peak) gradually declined. However, the green fluorescence intensity (green peak) gradually increased (Figure 4C), and the cells showed green fluorescence at the 2nd h (Figure 4B). The ratio of red/green fluorescence intensity gradually declined with prolonging treatment time, and it declined by 92.8% (*p* < 0.05) at the 2nd h (Figure 4D). These results suggested that the MMP in *C. reinhardtii* was seriously broken by norfloxacin.

### 2.5. Increase in Caspase-3-like Activity

**Figure 5:**
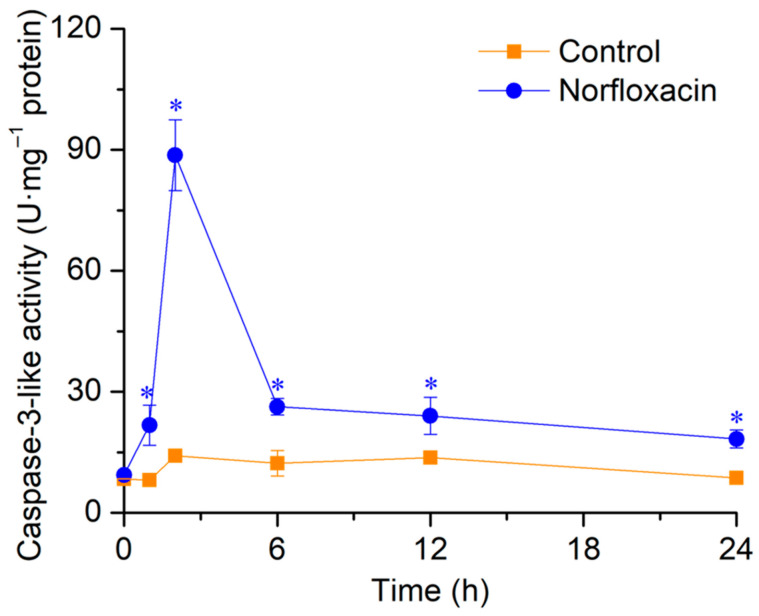
Effects of 50 μM norfloxacin on caspase-3-like activity in *C. reinhardtii*. *: Significant difference at *p* < 0.05 compared with the control (without norfloxacin treatment). Means ± SE (*n* = 4).

Without norfloxacin treatment, there was no remarkable variation in caspase-3-like activity in *C. reinhardtii* during the 24 h. In exposure to 50 μM norfloxacin, the enzyme activity reached the highest value after 2 h, with the increase of 10.6 folds (*p* < 0.05). After that, the activity gradually reduced, but was still remarkably higher than the control (Figure 5).

### 2.6. Changes in Cell Morphology

**Figure 6:**
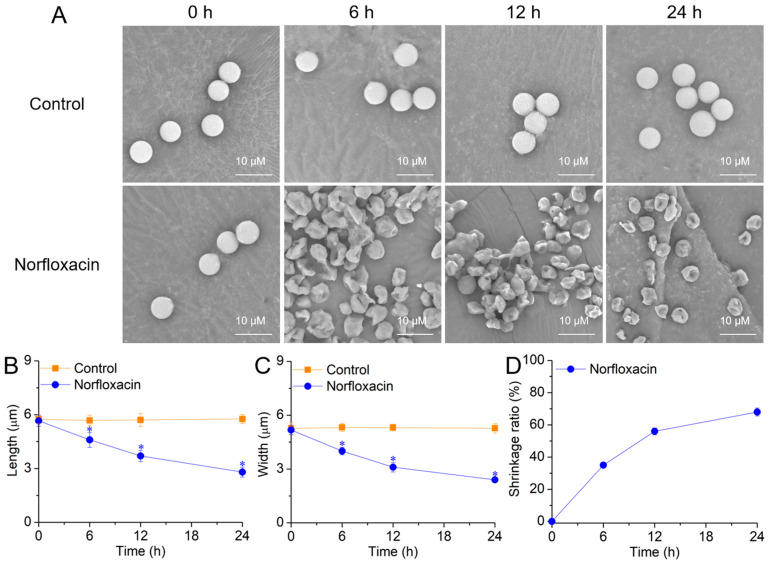
Effects of 50 μM norfloxacin on *C. reinhardtii* cell morphology. (**A**) Cell morphology; (**B**) Cell length; (**C**) Cell width; (**D**) Shrinkage ratio. *: Significant difference at *p* < 0.05 compared with the control (without norfloxacin treatment). Means ± SE (*n* = 4).

In exposure to 50 μM norfloxacin, *C. reinhardtii* cells showed a gradual shrinking and wrinkling trend during the 24 h treatment (Figure 6A). After 24 h, the cell length and width declined by 51.4% (*p* < 0.05) (Figure 6B) and 54.5% (*p* < 0.05) (Figure 6C), respectively. For the shrinkage ratio, it gradually increased and reached 68.0% after 24 h (Figure 6D).

### 2.7. Changes in TUNEL-Positive Nuclei

**Figure 7:**
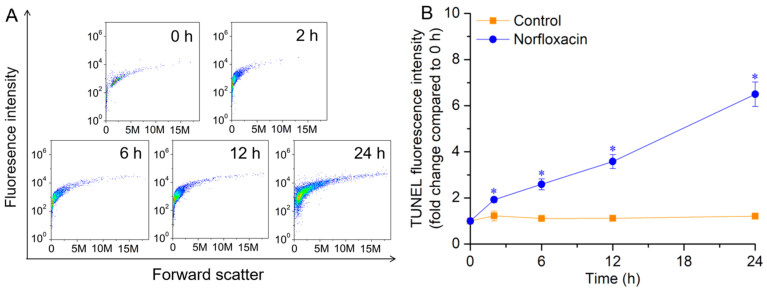
Effects of 50 μM norfloxacin on TUNEL-positive nuclei in *C. reinhardtii*. (**A**) The cells with TUNEL-positive nuclei detected by forward scatter. (**B**) Fluorescence intensity during 24 h. *: Significant difference at *p* < 0.05 compared with the control (without norfloxacin treatment). Means ± SE (*n* = 4).

During the 24 h treatment with 50 μM norfloxacin, the cell numbers of *C. reinhardtii* with TUNEL-positive nuclei gradually increased (Figure 7A). For the TUNEL fluorescence intensity, it also gradually enhanced with prolonging the treatment time, and increased by 5.4 folds (*p* < 0.05) after 24 h (Figure 7B).

### 2.8. DNA Laddering

**Figure 8:**
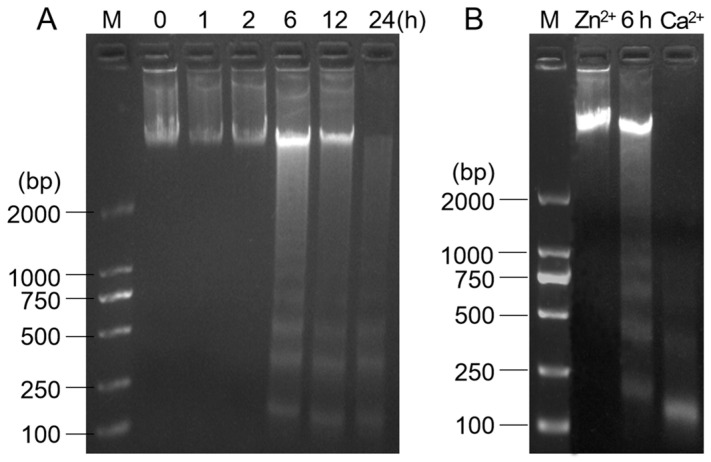
Effects of 50 μM norfloxacin on DNA ladders in *C. reinhardtii*. (**A**) DNA ladders during 24 h. (**B**) Inhibition and activation on DNA laddering. M: DNA marker. Zn^2+^ and Ca^2+^: *C. reinhardtii* was separately pretreated with 0.5 mM ZnSO_4_ and CaCl_2_ for 0.5 h and then treated with 50 μM norfloxacin for 6 h.

In the treatment with 50 μM norfloxacin, obvious DNA ladders were found after 6 h, and the cleavage gradually enhanced with prolonging the treatment time (Figure 8A). In Zn^2+^ pretreatment, the DNA degradation in *C. reinhardtii* treated with norfloxacin for 6 h was obviously inhibited, while an intensified effect was detected in Ca^2+^ pretreatment (Figure 8B).

## 3. Discussion

Norfloxacin is frequently used to kill bacteria by inhibiting their DNA replication and DNase activity [7], and has been widely found in water bodies due to discharge of wastewater containing the antibiotic [8]. Algae and cyanobacteria are essential primary producers in aquatic ecosystems, which are seriously poisoned by norfloxacin. In exposure to the antibiotic, the inhibition was found in the cell growth and photosynthesis in *M. aeruginosa* [17], *C. ovalisporum* [19] and *A. platensis* [20], and the inhibitory effect gradually aggravated with raising the concentration. For *M. aeruginosa*, *S. obliquus* and *Chlorella* sp., their ROS quickly burst and accumulated to high levels in the treatment with norfloxacin, which resulted in further oxidative stress to the cells [10,23,24,25]. For *S. quadricauda* and *S. obliquus*, their colony formation was impacted by norfloxacin [13,26]. Moreover, norfloxacin exhibited lethal effect on algae, and can kill *P. subcapitata* [30], *C. vulgaris* [31] and *S. obliquus* [25] cells directly. Similarly, norfloxacin also showed inhibitory and lethal effects on *C. reinhardtii*, which gradually intensified with increasing the concentration. In 50 μM norfloxacin treatment, the whole algal cells were dead after 24 h (Figure 1).

Cell death is the end of life and nonreversible termination of the life phenomenon, which includes two main patterns, such as passive death (necrosis) and active death (PCD) [36]. Necrosis is a rapid and uncontrolled death process, which is characterized by the rupture of plasma and nuclear membranes, disintegration of cytoskeleton and nuclear layers, as well as leakage of cellular contents [37,38]. However, PCD is quite different from necrosis, as it is an organized and controlled cell death, appearing with a series of hallmarks, such as MMP reduction, permeability transition pore (PTP) formation, cytochrome c (Cyt c) entering into cytosol, cell shrinkage, caspase activation, nuclear shrinkage and rupture, DNA cleavage and showing ladders [36,39].

When *C. reinhardtii* cells underwent PCD induced by triclosan, their photosynthetic abilities gradually reduced and ultimately disappeared [40]. The gradual disappearance of photosynthetic abilities and respiration was also found during the PCD in *C. reinhardtii* and *L. turionifera* caused by β-cyclocitral and β-ionone [36,41,42], and in *M. aeruginosa* induced by eucalyptol and borneol [37]. During the death process triggered by 50 μM norfloxacin, the photosynthetic and respiratory rate in *C. reinhardtii* decreased gradually, and disappeared after 24 h (Figure 3), demonstrating that the cell death might not be a necrosis but might be a PCD because of the gradual disappearance of the physiological activities.

ROS are unavoidably generated as the by-products in the respiratory electron transport chain in mitochondria, photosynthetic electron transport chain in chloroplasts, peroxisomes and cytoplasm, whereas stress conditions can enhance their generation [43,44,45,46]. In exposure to norfloxacin, the burst of ROS was detected in *Staphylococcus aureus* and *M. aeruginosa* cells in a short time [10,47]. In this study, the ROS were also quickly accumulated during norfloxacin-caused *C. reinhardtii* cell death, and their levels reached the highest value at the 2nd h (Figure 2). This was consistent with the quick accumulation and burst of ROS during *L. minor* PCD induced by ciprofloxacin [14] and *Arabidopsis thaliana* PCD triggered by 3 quinolone antibiotics [48], as well as H_2_O_2_ during *C. reinhardtii* PCD triggered by acetic acid, eucalyptol and β-cyclocitral [36,49].

ROS have strong oxidative capacity to cell membrane systems, and mitochondrial membranes are easily subjected to the oxidation, due to the organelle as one of the major ROS producers and primary targets [50]. The oxidative damage of mitochondrial membranes can improve the organelle permeability transition and lead to PTP formation, with lowering MMP [13]. When *Pseudomonas subcapitata* cells underwent PCD induced by N-(3-oxododecyl)-l-homoserine lactone, the mitochondrium depolarization and PTP formation were detected due to the massive accumulation of ROS [51]. When *C. reinhardtii* suffered PCD caused by ibuprofen and oxytetracycline, the accumulated ROS resulted in mitochondrium depolarization and MMP reduction [52]. Similar results were also detected during *C. luteoviridis* PCD triggered by paraquat [53], and *Argopecten irradians* PCD induced by several antibiotics such as sulfamethoxazole, tetracycline, oxytetracycline and erythromycin [54]. In this study, the MMP in *C. reinhardtii* cells was markedly declined in 50 μM norfloxacin treatment for 2 h, and it was gradually declined during the cell death (Figure 4), indicating that PTP have been formed in their mitochondria.

Following PTP opening, Cyt c enters into the cytosol from the mitochondria, and binds with Apaf-1 and caspase-9 to assemble a multimeric apoptosome complex that can activate the caspase cascade reaction to execute PCD [55]. In the cascade reaction, caspase-3 plays the final executive role [56]. In plants and algae, caspase-likes serve the same function of caspases in animal cells, and their activation is the crucial step in initiating PCD [42,57,58]. When *C. reinhardtii* suffered PCD induced by ibuprofen, the activation of caspase-3-like was found after 24 h [52]. Similarly, this activation was also found during the algal PCD induced by atrazine [59], triclosan [40] and benzophenone [60]. However, the PCD was remarkably inhibited after blocking caspase-3-like activity [36,41]. During norfloxacin-induced *C. reinhardtii* cell death, the activation of caspase-3-like was also found, and the enzyme activity increased to the maximum level after 2 h (Figure 5).

When algal cells undergo PCD, the contraction of cell morphology is detected as a common hallmark. For example, a shrinkage was found in *C. reinhardtii* suffering PCD caused by several compounds such as limonene, β-ionone, longifolene and mastoparan [42,61], as well as stresses of UV irradiation and KCl [62,63]. The similar shrinkage was also detected in *M. aeruginosa* PCD induced by H_2_O_2_, eucalyptol and borneol [37,64,65], and in *C. saccharophila* PCD caused by heat stress [66]. In this study, the shrinkage and wrinkle were also found in *C. reinhardtii* cell death caused by norfloxacin and showed a gradual intensifying trend during the death process (Figure 6).

During PCD, nuclear DNA are cut into small fragments by endonucleases and expose abundant free 3′-OH ends that can bind with TUNEL dyes to show fluorescence [37,41]. When *C. reinhardtii* PCD was induced by acetic acid at pH 5.0, the numbers and fluorescence intensity of TUNEL-positive nuclei gradually increased during the death process [49]. Similarly, the positive nuclei also appeared in *A. thaliana* root PCD triggered by low pH [67], wheat root PCD triggered by aluminum stress [68], and *L. turionifera* PCD caused by β-cyclocitral and β-ionone [41]. *M. aeruginosa* is a cyanobacterium, without cell nucleus. Then, the whole cell showed bright TUNEL fluorescence during borneol- and eucalyptol-triggered PCD [37]. In this study, the TUNEL-positive nuclei were also found in norfloxacin-caused *C. reinhardtii* death, and their numbers and fluorescence intensity gradually enhanced during the death process (Figure 7).

Among PCD hallmarks, DNA laddering is considered as the typical one [36,41]. When human corneal epithelial cells were treated with norfloxacin, an obvious DNA laddering was detected during the cells undergoing PCD [69]. The DNA laddering was also found in corneal endothelial cell PCD induced by norfloxacin [70]. For *C. reinhardtii* cells, their DNA were cleaved into small fragments and showed ladders in electrophoretic bands during the PCD induced by several cyanobacterial compounds, such as longifolene, β-ionone, limonene, eucalyptol and β-cyclocitral, and the laddering gradually enhanced during the process [36,42]. In exposure to 50 μM norfloxacin for 6 h, obvious DNA ladders were found in *C. reinhardtii*, and the laddering gradually intensified with prolonging the treatment time (Figure 8A).

During PCD, the DNA ladders are generated by breaking the phosphodiester bonds in catalysis with Ca^2+^-dependent endonucleases [71]. It has been reported that Zn^2+^ can efficiently inhibit the activity of Ca^2+^-dependent endonucleases associated with animal and plant PCD [72], while Ca^2+^ shows activation effect [73,74]. In Zn^2+^ pretreatment, the DNA degradation and ladder formation were blocked during *C. reinhardtii* PCD caused by acetic acid, eucalyptol and β-cyclocitral [36,49], *M. aeruginosa* PCD induced by eucalyptol and borneol [37], and *L. turionifera* PCD induced by β-cyclocitral and β-ionone [41]. For *Oncorhynchus mykiss*, Zn^2+^ pretreatment inhibited the DNA degradation during the PCD, while Ca^2+^ pretreatment accelerated the formation of DNA laddering [74]. In norfloxacin-induced *C. reinhardtii* death, Zn^2+^ and Ca^2+^ also showed inhibitory and accelerated effects on the formation of DNA laddering, respectively, demonstrating that Ca^2+^-dependent endonucleases played the cleaving role (Figure 8B).

## 4. Materials and Methods

### 4.1. Norfloxacin Treatment

*C. reinhardtii* strain CC400 were provided by Prof. Cuimin Liu of the Institute of Genetics and Developmental Biology, Chinese Academy of Sciences, China, and grown in tris-acetate-phosphate (TAP) medium [75] with a light intensity of 50 μmol·m^−2^·s^−1^ in a 16 h light and 8 h dark cycle at 25 °C. The shaker speed was set as 90 rpm. Once the algal cell density reached the logarithmic growth stage, the cells were collected by centrifugation and transferred into conical flasks with 25 mL fresh TAP medium (5 × 10^6^ cells·mL^−1^) under sterile conditions. An aliquot of a 5 mM norfloxacin stock solution was added to treat the algal cells, with the concentration of 10, 30 and 50 μM, respectively, while the algal cultures with adding the same amount of distilled water (without norfloxacin) were used as the control. Four replicates were set in each treatment, with each conical flask as a replicate. The cell growth, dead ratio, ROS levels, photosynthetic and respiratory rate, MMP, caspase-3-like activity, cell morphology, TUNEL-positive nuclei and DNA ladders were determined during the 24 h treatment.

### 4.2. Assay of Cell Growth and Dead Ratio

*C. reinhardtii* cells were stained with 0.2‰ neutral red, of which live and dead (showing red color) cell numbers were counted using a light microscope at 200× magnification (Olympus CX31, Tokyo, Japan) with a 25 × 16 hemocytometer. Then, the live cell numbers were used to calculate the cell density, while the dead cell numbers were used to calculate the dead ratio by dividing by the total (alive + dead) cell numbers [37].

### 4.3. Assessment of ROS Levels

The algal cells were collected from 10 mL cultures by centrifugation, and their ROS levels were assessed by the probe 2,7-dichlorodihydrofluorescein diacetate (DCFH-DA) according to the previous method [76]. After entering the cells, DCFH-DA was hydrolyzed to generate 2′,7′-dichlorodihydrofluorescein, and the formed compound was oxidized by ROS to generate the fluorescent 2′,7′-dichlorofluorescein (DCF). Then, the fluorescence (about 530 nm) was observed and measured by using a fluorescence microscope, magnifying 400 times (Olympus BX51, Tokyo, Japan), and flow cytometer (BD Accuri™ C6 Plus, Franklin Lakes, NJ, USA), respectively.

### 4.4. Determination of Photosynthetic and Respiratory Rate

Twenty mL *C. reinhardtii* cultures were poured into the cup of an oxygen electrode (YZQ Technology Co., Beijing, China), and the bubbles were eliminated. The O_2_ evolution rate was measured at 25 °C under the light (700 μmol·m^−2^·s^−1^) provided by LEDs to indicate the photosynthetic rate [77], while the O_2_ consumption rate was determined at 25 °C in darkness to indicate the respiratory rate [37].

### 4.5. MMP Detection

MMP was detected using an assay kit with 5,5′,6,6′-tetrachloro-1,1′,3,3′ tetraethylbenzimidazolyl-carbocyanine iodide (JC-1) (Beyotime Biotechnology, Shanghai, China). Twenty-five mL algal cultures were centrifuged at 6000 *g*, and the collected cells were washed twice using 10 mM phosphate buffer solution (PBS). Then, they were resuspended in 2 μM JC-1 solution of 500 μL. For healthy mitochondria, JC-1 enters into and aggregates in the matrix to form polymers with releasing red fluorescence (about 590 nm). Otherwise, the compound lies in the cytoplasm as a monomer releasing green fluorescence (about 530 nm) when mitochondria are damaged, with membrane potential decrease or loss. After incubation at 37 °C for 0.5 h following the protocol of the kit, the cells were centrifuged at 4 °C, washed three times with PBS, and resuspended in 500 μL PBS. A fluorescence microscope (magnifying 400 times) and flow cytometer were separately used to observe and measure the fluorescence, and the ratio of red/green fluorescence intensity was calculated to indicate the MMP variation.

### 4.6. Determination of Caspase-3-like Activity

Twenty-five mL algal cultures were centrifugated at 6000 *g*, and the collected cells were lysed by adding into 100 μL lysis buffer. The lysis solution was centrifuged at 4 °C, of which supernatants were added into 100 μM Ac-DEVD-pNA, the substrate of caspase-3. Then, the mixture was kept at 37 °C for 2 h, of which absorption at 405 nm was measured. Following the previous method, the plot of the absorption at 405 nm and gradient pNA concentration was used to draw the standard curve [41]. In the extracts, the protein levels were also determined as described by Peng et al. (2020) [78]. During the enzymatic reaction, the yield of 1 nM pNA was considered as a unit of caspase-3-like activity, and the enzyme activity per mg protein was evaluated.

### 4.7. Observation of Algal Cell Morphology

The algal cultures were centrifuged at 6000 *g*, and the cells were fixed in double aldehyde solution (2.5% glutaraldehyde: 40% formaldehyde, 1:1) at 4 °C for 6 h. After dehydration, the dried cells were sprayed with platinum, and then their morphology was observed by using a scanning electron microscope (Phenom Pro, Almelo, The Netherlands). In a field, the cell length, width and area were determined with ImageJ (National Institutes of Health, Bethesda, MD, USA), and the cell shrinkage ratio was assessed following the formula shrinkage ratio = (A_C_ − A_T_)/A_C_ × 100%, where A_C_ and A_T_ are the area of the control and treatment, respectively.

### 4.8. TUNEL Assay

After centrifugation, *C. reinhardtii* cells were fixed in 4% formaldehyde solution, and incubated with TUNEL fluorescein for 1 h. For broken DNA, the exposed 3′-OH can combine with the fluorescein in the catalysis of terminal deoxynucleotidyl transferase in the cells. Then, the complex showed green fluorescence at about 530 nm, of which intensity was detected using a flow cytometer.

### 4.9. DNA Laddering Assay

Following our previous method [49], the harvested algal cells after centrifugation were resuspended in 400 μL NET solution (100 mM NaCl, 50 mM EDTA, and 20 mM Tris-HCl). Then, 50 μL 100 g·L^−1^ SDS and 13 μL 20 g·L^−1^ proteinase K were added into the suspension for lysing the cells. The DNA was extracted from the lysate by using chloroform-isoamyl alcohol and precipitated by using cold ethanol. When the RNA was degraded with 10 mg·mL^−1^ DNAse-free RNAse, approximately 5 μg DNA was used to detect the DNA ladders through agarose gel electrophoresis [42].

Zn^2+^ and Ca^2+^ are separately the inhibitor and activator of Ca^2+^-dependent endonucleases that can cause DNA degradation. To uncover whether the endonucleases took charge of the DNA ladders, *C. reinhardtii* cells were incubated with 0.5 mM ZnSO_4_ and CaCl_2_, respectively. After 0.5 h, they were treated with 50 μM norfloxacin for 6 h, and the DNA laddering was assayed as described in the above method.

### 4.10. Statistical Analysis

All statistical analyses were performed using Origin 8.5 (Origin Lab, Northampton, USA). An independent-samples *t*-test was used to compare the differences (*p* < 0.05) between the treatment and the control. Prior to the *t*-test, the assumptions of normality and homogeneity of variances were assessed. Normality was evaluated using the Shapiro–Wilk test for each group, and homogeneity of variances was verified using Levene’s test.

## 5. Conclusions

Norfloxacin had a toxic effect on *C. reinhardtii* and even killed the whole of the cells at 50 μM. During the cell death, the photosynthesis and respiration gradually disappeared, whereas ROS quickly burst and was maintained at high levels all the time. This death process can be evidenced to be a PCD by the appearance of remarkable hallmarks, including MMP reduction, caspase-3-like activation, cell shrinkage, TUNEL-positive nuclei as well as DNA laddering. In the process, Ca^2+^-dependent endonucleases may cleave the DNA to form ladders. It can be speculated that norfloxacin can poison algae by causing PCD triggered by the induced ROS, which may seriously disrupt the aquatic ecosystem due to the lethal effect on the primary producers.

## Data Availability

The original contributions presented in this study are included in the article.
